# Synthesis, Characterization and Microstructure of New Liquid Poly(methylhydrosiloxanes) Containing Branching Units SiO_4/2_

**DOI:** 10.3390/polym10050484

**Published:** 2018-04-28

**Authors:** Jerzy J. Chruściel, Marzena Fejdyś, Witold Fortuniak

**Affiliations:** 1 Textile Research Institute, Brzezińska Str. 5/15, 92-103 Łódź, Poland; 2 The Institute of Security Technologies “MORATEX”, Marii Skłodowskiej-Curie Str. 3, 90-505 Łódź, Poland; mfejdys@moratex.eu; 3 Centre of Molecular and Macromolecular Studies, Polish Academy of Sciences, Sienkiewicza Str. 112, 90-363 Łódź, Poland; wfortuni@cbmm.lodz.pl

**Keywords:** hyperbranched poly(methylhydrosiloxanes), hydrolytic polycondensation, ^29^Si-NMR, topology of polysiloxane chains

## Abstract

Six liquid branched poly(methylhydrosiloxanes) of new random structures (PMHS-Q), containing quadruple branching units SiO_4/2_ (Q), both MeHSiO (D^H^) and Me_2_SiO (D) chain building units (or only mers MeHSiO), and terminal groups Me_3_SiO_0.5_ (M) were prepared by a hydrolytic polycondensation method of appropriate organic chlorosilanes and tetraethyl ortosilicate (TEOS), in diethyl ether medium at temperature below 0 °C. Volatile low molecular weight siloxanes were removed by a vacuum distillation at 150–155 °C. Yields of PMHS-Q reached from 55–69 wt%. Their dynamic viscosities were measured in the Brookfield HBDV+IIcP cone-plate viscometer and ranged from 10.7–13.1 cP. Molecular weights (MW) of PMHS-Q (M_n_ = 2440–6310 g/mol, M_w_ = 5750–10,350 g/mol) and polydispersities of MW (M_w_/M_n_ = 2.0–2.8) were determined by a size exclusion chromatography (SEC). All polymers were characterized by FTIR, ^1^H- and ^29^Si-NMR, and an elemental analysis. A microstructure of siloxane chains was proposed on a basis of ^29^Si-NMR results and compared with literature data.

## 1. Introduction

Poly(methylhydrosiloxanes) (PMHS) are inorganic–organic hybrid polymers with inorganic backbone, composed of alternatively bound silicon and oxygen atoms. Hydrogen atoms and methyl groups are the main substituents of silicon atoms in PMHS; however, other organic groups may be attached to silicon atoms in their structures as well. Most PMHS are colorless oils, though some of them are solids [1,2,3,4,5,6,7,8,9]. Linear, star, hyperbranched and dendritic poly(methylhydrosiloxanes), as well as spherical hydrosilicates, are important classes of functional silicones. Many methods of their preparation were described in our previous publication, concerning synthesis and characterization of branched PMHS containing triple branching units MeSiO_1.5_ (T) [9]. PMHS find numerous practical applications. Most often they are used as cross-linking agents in a technology of silicone elastomers [9,10,11,12,13,14,15].

One of the newer synthetic methods used for the preparation of poly(dimethylsiloxanes- -*co*-methylhydrosiloxanes) (PDMS-*co*-PMHS) is based on an equilibration polymerization of poly(dimethylsiloxanes) with linear hydrosiloxane polymer and hexamethyldisiloxane Me_3_SiOSiMe_3_, towards phosphonitrile catalyst [Cl_3_P(NPCl_2_)_2_PCl_3_]^+^PCl_6_^−^ at elevated temperature [16,17,18,19,20].

A polymerization of 1,3,5,7-tetramethylcyclotetrasiloxane (D^H^_4_) [D^H^ = H(CH_3_)SiO] was carried out in the presence of the non-ionic emulgator C_12_H_25_(OCH_2_CH_2_)_23_OH and dodecylbenzene- sulphonic acid and it was accelerated by ultrasounds [21,22].

Weber and Paulasaari obtained poly(1-hydro-1,3,3,5,5-pentamethyltrisiloxane) with a regular chain structure by the polymerization of a new monomer, pentamethylcyclotrisiloxane c-(Me_2_SiO)_2_(MeHSiO) [c-D_2_D^H^; D = (CH_3_)_2_SiO] in tetrahydrofurane (THF), in the presence of Ph_2_Si(OLi)_2_, at −79 °C [23]. The starting monomer c-D_2_D^H^ was prepared by heterocondensation of MeHSiCl_2_ with HO(Me_2_SiO)_2_H (yield: 49%). At the same time was elaborated a new sequential polycondensation method (“one-pot”) leading to three homological series of new PDMS-*b*-PMHS with a general formula:

RMe_2_SiO[(Me_2_SiO)_m_(MeHSiO)_k_]_n_(Me_2_SiO)_m_SiMe_2_R, (1)

(where: R = –OH or –Me; m = 2, 6, 10, 14, ~50; k = 1–4; n = 5, 10, 15, 20), containing single or multiple MeHSiO units in macromolecules [9,24]. This synthetic method is based on a non-stoichiometric polycondensation of appropriate dimethylsiloxane-α,ω-diols HO(Me_2_SiO)_m_H with siloxane oligomers of a general formula Cl(MeHSiO)_k-1_SiHMeCl, having chloro(hydro)silyl functionalities, followed by termination reactions with chlorotrimethylsilane Me_3_SiCl, when PDMS-*b*-PMHS chains were blocked with (CH_3_)_3_SiO_0.5_ (M) groups. An isolation and characterization of many model H-siloxanes helped us to establish the regular microstructure of prepared PDMS-*b*-PMHS, which was further confirmed by ^1^H- and ^29^Si-NMR studies [9,25].

In recent years a growing interest has been observed in the field of syntheses of star, highly branched, and dendritic poly(methylhydrosiloxanes). Cage silsesquioxanes (“spherosiloxanes”): T^H^_8_, T^H^_10_, T^H^_12_, T^H^_14_, and T^H^_16_ (T^H^ = HSiO_1.5_) are solids, which can be prepared by the hydrolytic polycondensation of: (a) trichlorosilane HSiCl_3_, carried out in the presence of FeCl_3_ in methanol medium [24,26,27,28], or (b) trimethoxysilane HSi(OCH_3_)_3_, saturated with anhydrous HCl, in acetic acid solution [29,30,31,32], or in the presence of concentrated H_2_SO_4_ [33,34]. Octakis(dimethylsiloxy)- octasilsesquioxane [(HMe_2_SiO)SiO_1.5_]_8_ [Q_8_M^H^_8_, Q = SiO_4/2_, M^H^ = H(CH_3_)_2_SiO_0.5_] can be prepared with high yields in reaction of octakis(tetramethylammonium)octasilsesquioxane with ClSiMe_2_H [35,36,37], up to 85–91% yield [38,39]. Currently a low molecular mass star tetrakis(dimethylsiloxy)silane Si[OSi(CH_3_)_2_H]_4_, octahydrosilsesquioxane (T^H^_8_) and cubic Q_8_M^H^_8_ are commercially available [35,40,41,42,43,44,45,46].

Recently siloxane-polyhedral silsesquioxane copolymers (soluble in THF) were prepared by the dehydrogenative condensation of T^H^_8_ with diphenylsilanediol, tetraphenyldisiloxane diol or oligodimethylsiloxane-α,ω-diols in the presence of diethylhydroxylamine, followed by trimethyl- silylation [47,48]. T^H^_8_ was also applied as a precursor of mezoporous silica, which was prepared without using any template or surfactant [49].

An equilibration of octamethylcyclotetrasiloxane [(Me_2_SiO)_4_, D_4_] with Si[OSi(CH_3_)_2_H]_4_ and trifluoromethanesulphonic acid led to tetraarm star polysiloxane Si{[OSi(CH_3_)_2_]_n_OSi(CH_3_)_2_H}_4_ [50,51]. Six- and eight-membered silicates: hexakis(dimethylsiloxy)cyclotrisiloxane [(HMe_2_SiO)_2_SiO]_3_ and octakis(dimethylsiloxy)cyclotetrasiloxane [(HMe_2_SiO)_2_SiO]_4_ were synthesized with low yields, from reactions of pyrolysis products of wollastonite. [(HMe_2_SiO)_2_SiO]_3_ was prepared in reaction of chlorodimethylsilane HMe_2_SiCl with pseudowollastonite Ca_3_Si_3_O_9_Cl_6_, while [(HMe_2_SiO)_2_SiO]_4_ was prepared by heating octakis(trimethylsiloxy)cyclotetrasiloxane [(Me_3_SiO)_2_SiO]_4_ with 1,1,3,3- -tetramethyldisiloxane HMe_2_SiOSiMe_2_H in the presence of trifluoromethanesulphonic acid [52,53]. The equilibration of [(HMe_2_SiO)_2_SiO]_3_ with D_4_ and triflic acid gave PMHS of the following branched structure: –{–OSi[(OSiMe_2_O)_n_SiMe_2_H]_2_–}_6_– [52]. The Si–H terminated multifunctional silicone dendrimer, i.e., tetrakis(dimethylsiloxy)silane, was prepared with 69% yield by the reaction of TEOS and dimethoxysilane [54].

Zhang et al. synthesized polysilsesquioxanes of a ladder structure, containing units HSiO_1.5_ and MeSiO_1.5_, by hydrolysis of byproducts prepared through condensation of HSiCl_3_ and MeSiCl_3_ with *p*-phenylenediamine or ethylenediamine [55,56,57].

A silicone dendrimer of a third generation with symmetrical structure and the general formula (CH_3_SiO_1.5_)_22_[(CH_3_)_2_SiO)]_162_[H(CH_3_)_2_SiO_0.5_]_24_ was prepared by Masamune et al. [58] in a multistep synthesis from siloxane oligomers containing functional groups: Si-H, Si-Cl, Si-Br and Si-OH. It had 24 terminal functional Si-H groups “on the surface”. Branched resins, containing 10–14 Si-H functional groups in macromolecules were synthesized by the hydrolytic polycondensation of methyldichlorosilane with dimethyldichlorosilane, trimethylchlorosilane and methyltriethoxy)- silane or phenyl(triethoxy)silane. These resins were used as crosslinking agents for addition of cured silicone elastomers [59]. Condensation of (triethoxy)silane HSi(OC_2_H_5_)_3_, towards HCl solution, in the mixture of THF and methylisobutyl ketone (MIBK), led to soluble multifunctional poly- (hydrogensilsesquioxanes) (PHSSQ) of combined cage-like and network-like structures [60]. A solid four-membered silsesquioxane ring compound (PhSiO_1.5_)_8_(MeHSiO)_2_, so-called ”double-decker- shaped-silsesquioxane”, was prepared from reaction of MeHSiCl_2_ with a byproduct, which was obtained via a condensation of phenyl(trimethoxy)silane and NaOH with 20% yield [61].

Twelve new liquid branched poly(methylhydrosiloxanes) with statistical structures (b-r-PMHS), containing triple branching units MeSiO_1.5_ (T), both Me_2_SiO (D) and MeHSiO (D^H^) chain building units (or only mers MeHSiO), and two b-r-PMHS containing five different structural units: D, D^H^, T and T^H^ and trimethylsiloxy end groups Me_3_SiO_0.5_ (M) were prepared by the hydrolytic poly- condensation method of appropriate chlorosilanes in diethyl ether medium at temperature <0 °C. Yields of b-r-PMHS ranged from 57–84 wt% (after removal of low molecular weight oligosiloxanes by a vacuum distillation at 125–150 °C). All polymeric products were characterized by FTIR, ^1^H- and ^29^Si-NMR, and elemental analysis. Their dynamic viscosities were very low and usually ranged from ~8–30 cP, which presumably resulted from their globular structure [9].

Methyl-substituted silica gels with Si-H functionalities were prepared by hydrolysis and condensation reactions of triethoxysilane and methyldiethoxysilane, used in various molar ratios [62]. They gave higher ceramic residue after pyrolysis than gels based only on MeSiO_1.5_ branching units [63].

In the present work, we describe the hydrolytic polycondensation synthetic route to new liquid branched poly(methylhydrosiloxanes) of random structures (PMHS-Q), containing both MeHSiO (D^H^) and Me_2_SiO (D) chain building units (or only mers MeHSiO), quadruple branching units SiO_4/2_ (Q), and terminal groups Me_3_SiO_0.5_, from appropriate organic chlorosilanes and tetraethoxysilane.

## 2. Materials and Methods

Dichloromethylsilane MeHSiCl_2_ (MDS, 99%, b.p. 41 °C), dichlorodimethylsilane Me_2_SiCl_2_ (DDS, b.p. 70–71 °C), tetraethoxysilane Si(OC_2_H_5_)_4_ (b.p. 168 °C), (4-dimetylamino)pyridine (DMAP, 99%) were all sourced from Aldrich Chemical Company Inc., USA. Chlorotrimethylsilane Me_3_SiCl was obtained from Fluka, Seelze, Germany (TMCS, >99%, b.p. 57 °C). Tetraethoxysilane Si(OEt)_4_ was obtained from Unisil, Tarnów, Poland (TEOS, 99%, b.p. 168 °C). Triethylamine (>99%, Fluka) was dried with anhydrous KOH, decanted, and distilled over P_2_O_5_. Diethyl ether was purified and dried with anhydrous KOH, and distilled over CaH_2_.

All products were analyzed by a nuclear magnetic resonance (NMR), infrared spectroscopy (FTIR) and gel chromatography (SEC). FTIR spectra (neat) were done on spectrophotometer IR Bio-Rad 175 C (American Laboratory Trading, East Lyme, CT 06333, USA) for samples placed between NaCI plates. ^1^H-NMR and ^29^Si-NMR (INEPT) spectra were recorded on Bruker DRX 500 machine (Bruker Physik AG, Karlsruhe, Germany) at CBMM PAN in Łódź. Hexamethyldisiloxane Me_3_SiOSiMe_3_ was used as an external standard in ^29^Si-NMR (δ = 6.98 ppm, in CDCl_3_).

An elementary analysis (% C and % H) was performed at the Centre of Molecular and Macromolecular Studies of the Polish Academy of Sciences in Łódź (CBMM PAN). The content of Si-H groups was calculated from an integration ratio of their signals to CH_3_ signals in ^1^H-NMR spectra, and compared to theoretical integration ratios of Si-H and CH_3_ signals. The content of Si was determined by the gravimetric method with H_2_SO_4_ (p.a.) [64].

Dynamic viscosities (η^25^) of polysiloxanes were measured at 25.0 °C in a Brookfield cone-plate reoviscometer HBDV-II+cP (Brookfield Engineering Laboratories, Inc., Middleboro, MA 02346, USA), using a cone cP40.

The molecular masses and molecular mass distribution of polysiloxanes were analyzed by a size exclusion chromatography (SEC) in toluene solution, using LDC analytical chromatograph (Artisan Technology Group, Champaign, IL 61822, USA) equipped with refractoMonitor and a battery of two phenogel columns covering the MW range 10^2^–10^5^ g∙mol^−1^. Calibration was made with polystyrene Ultrastyrogel standards with MWs: 10^2^, 10^3^, and 10^4^ g∙mol^−1^.

### Synthesis of Branched Polymethylhydrosiloxanes (PMHS-Q)

Branched polymethylhydrosiloxanes, containing only units D^H^ and Q, terminated with Me_3_SiO_0.5_ groups, with structures described by a general formula:

(SiO_4/2_)_y_[CH_3_(H)SiO]_n_[(CH_3_)_3_SiO_0.5_]_p_ (2)

(where: y = 1–3, n = 48–50, p = 2y + 2), and branched poly(dimethyl-*co*-methylhydro)siloxanes, containing both mers D, as well mers D^H^, units Q and end Me_3_SiO_0.5_ groups, of a general formula:

(SiO_4/2_)_y_[(CH_3_)_2_SiO]_m_[CH_3_(H)SiO]_n_[(CH_3_)_3_SiO_0.5_]_p_ (3)

(where: y = 1–3, m = n = 49–52, p = 2y + 2), were synthesized by the hydrolytic polycondensation of mixtures of tetraethoxysilane Si(OEt)_4_ and appropriate chlorosilanes: dichloromethylsilane MeHSiCl_2_, dichlorodimethylsilane Me_2_SiCl_2_, and chlorotrimethylsilane Me_3_SiCl, in the medium of diethyl ether and water, at temperature ranged from −10–0 °C, within 3–5 h. Molar ratios of chlorosilanes were changed, depending on expected molecular formula of polysiloxane. Amounts of substrates used in syntheses of branched PMHS-Q and times of additions of chlorosilanes are presented in Table 1. In the hydrolytic polycondensation reactions were used such amounts of distilled water, which were sufficient for a formation of hydrochloric acid with a final concentration about 20 wt%.

Reaction mixture was allowed to warm to room temperature within 120–170 min, acid layer was separated, and organosilicon layer was washed with water until neutral, transferred to an Erlenmayer flask, and dried at ~4 °C with anhydrous magnesium sulfate overnight. Magnesium sulfate was filtered through Schott funnel G-3 and washed with ether. Alternatively, instead of drying with anhydrous MgSO_4_ traces of water were removed from products by cooling their ether solution in a refrigerator overnight, warming up the content of the flask to room temperature, and the ether solution of products was decanted from drops of water. The solvent was distilled off. In order to remove volatile cyclic and linear low molecular weight siloxane oligomers, the prepared products were heated at temperature 150–155 °C under reduced pressure (16–21 mm Hg, 2128–2793 Pa), and subsequently under a vacuum (3–5 mm Hg, 400–665 Pa).

In a second step of syntheses of **Q_3_D^H^_50_M_8_** and other poly(dimethyl-*co*-methylhydro)siloxanes, containing both mers D and D^H^, with a general formula:

(SiO_4/2_)_y_[(CH_3_)_2_SiO]_m_[CH_3_(H)SiO]_n_[(CH_3_)_3_SiO_0.5_]_p_ (3)

(where: y = 1–3, m = n = 49–52, p = 2y + 2), so called “extra blocking” of unreacted silanol groups Si-OH was applied: in reactions with (chloro)trimethylsilane, in the presence of triethylamine, which was used as an acceptor of hydrogen chloride with ~5% excess with respect to a stoichiometric amount. (4-Dimethylamino)pyridine (DMAP) was used as a nucleophilic catalyst in 1:10 mole ratio with respect to Et_3_N. Products untreated with extra amounts of TMCS and DMAP/Et_3_N showed increase of their viscosity after few months and a presence of small drops of water from a homo- condensation reaction of residual Si-OH groups.

The “extra blocking” reactions of silanol groups were carried out after drying step of ether solutions of products of the hydrolytic polycondensation, at room temperature within few hours. Precipitates of amines hydrochlorides were dissolved in diluted solution (5–10 wt%) of hydrochloric acid, a water layers were discarded and washed with distilled water until neutral, dried with anhydrous MgSO_4_, and filtered. Ether was distilled off under atmospheric pressure and final products were evacuated under vacuum at temperature 150–155 °C (Table 2). A chemical composition of volatile siloxanes was not analyzed.

## 3. Results and Discussion

### 3.1. Synthesis of Branched Polymethylhydrosiloxanes (PMHS-Q)

Syntheses of poly(methylhydrosiloxanes) with statistical and branched structures containing quadruple branching points SiO_4/2_ were carried out in the medium of diethyl ether at temperature below 0 °C. Solutions of chlorosilanes and Si(OEt)_4_ in dry ether were added dropwise to water. In all syntheses were used such amounts of water which were necessary for hydrolysis reactions and dissolution of HCl, allowing to obtain hydrochloric acid with concentrations approximately 20 wt%.

Applying the hydrolytic polycondensation of mixtures of appropriate amounts of (tetraethoxy)-silane Si(OEt)_4_ and chlorosilanes: MeHSiCl_2_, Me_2_SiCl_2_, and Me_3_SiCl, with water, at temperature from −10–0 °C, within 3–5 h, were prepared branched poly(methylhydrosiloxanes) with SiO_4/2_ branching points and the general formula:

[SiO_4/2_]_y_[CH_3_(H)SiO]_n_[(CH_3_)_3_SiO_0.5_]_p_ (2)

(where: y = 1–3, n = 48–50, p = 2y + 2), containing quadruple branching points SiO_4/2_ (Q), mers D^H^ and terminal groups Me_3_SiO_0.5_. Similarly, branched poly(dimethyl-*co*-methylhydro)siloxanescontaining branching units Q, linear building blocks D, and D^H^, and terminal groups M, were synthesized with the general formula:

[SiO_4/2_]_y_[(CH_3_)_2_SiO]_m_[CH_3_(H)SiO]_n_[(CH_3_)_3_SiO_0.5_]_p_ (3)

where y = 1–3, m = n = 49–52, p = 2y + 2. After addition of substrates stirring of obtained reaction mixtures was continued within next 2–3 h, in order to reach full conversion of substrates and full hydrolysis of Si-Cl and Si-OC_2_H_5_ groups. In the case of syntheses of **Q3**, **Q1D**, **Q2D**, and **Q3D** termination reactions (so called “extra blocking” reactions) of unreacted silanol groups Si-OH in reactions with (chloro)trimethylsilane were applied, in the presence of: (1) triethylamine as the acceptor of hydrogen chloride (used with ~5–10% excess with respect to stoichiometric amounts); and (2) (4-dimethylamino)pyridine (DMAP) as the nucleophilic catalyst (used in 1:10 mole ratio with respect to Et_3_N).

Products not treated with additional amounts of TMCS and DMAP/Et_3_N showed increase of their viscosity after few months and a presence of traces of water, which could originate from the homocondensation reaction of residual Si-OH groups. However, in the case of syntheses of **Q1** and **Q2** “extra blocking” was not applied, and no increase of their viscosity was observed during longer storage of these PMHS-Q. Ether solutions of products **Q1**, **Q2**, and **Q3** were dried with anhydrous MgSO_4_, while polymers **Q1D**, **Q2D**, and **Q3D** were dried by freezing traces of water in the refrigerator overnight. Yields of prepared PMHS-Q ranged from 55–69 wt% (Table 2). The highest yield was obtained for **Q3**.

The chemical structures of all PMHS-Q were confirmed by spectroscopic methods: FTIR and NMR (^1^H and ^29^Si) and the elemental analysis (% C, % H, and % Si) (see Table 3).

Dynamic viscosities (η^25^) of PMHS-Q containing quadruple branching points SiO_4/2_, were very low and ranged from 10.7–13.1 cP. Low viscosities of PMHS-Q in comparison with linear polysiloxanes having similar molecular weights presumably may result from a globular structure of hyperbranched macromolecules. It is commonly known from a literature that dendrimers and hyperbranched polymers in solution and in melt have low viscosities. Their viscosities and molecular weights are much lower than those for linear analogs and depend on a degree of branching, a polarity of a solvent, a kind of functional group on their “surface”, and also on pH of a polymer solution. Dendritic and hyperbranched polymers have a variable hydrodynamic radii depending on the property of solvents; they are smaller than those of their linear analogs with the same molar mass.

The values of molecular weights of prepared PMHS-Q determined by SEC method were lower than calculated values for predicted molecular formulas: QD_52_D^H^_52_M_4_, Q_2_D_49_D^H^_49_M_6_, and Q_3_D_50_D^H^_50_M_8._ A polydispersity of molecular weights of PMHS-Q ranged from 2.0 to 2.8. The molecular weights of dendrimers and hyperbranched polymers determined by SEC using polystyrene standards are regarded with some scepticism. The hydrodynamic radii were also susceptible to the polarity of functional groups on the periphery [65,66,67]. Values of M_n_ and M_w_ determined by SEC method with polystyrene standards for hyperbranched polysiloxanes were much lower than MW obtained with application of MALLS detectors [68,69,70].

Köhler et al. used the SEC, ^1^H- and ^29^Si NMR, and MALDI-TOF-MS methods for characterization of a linear poly(dimethylsiloxane)-*co*-poly(hydromethysiloxane) (PDMS-*co*-PHMS) copolymer with respect to chain length distribution, heterogeneity of chemical composition, and sequence distribution [71].

### 3.2. Characterization of PMHS-Q by FTIR

**Figure 1:**
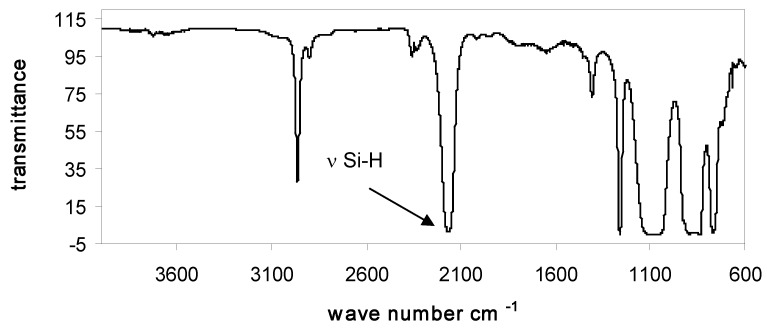
FTIR spectrum of Q_2_D^H^_49_M_6_.

**Figure 2:**
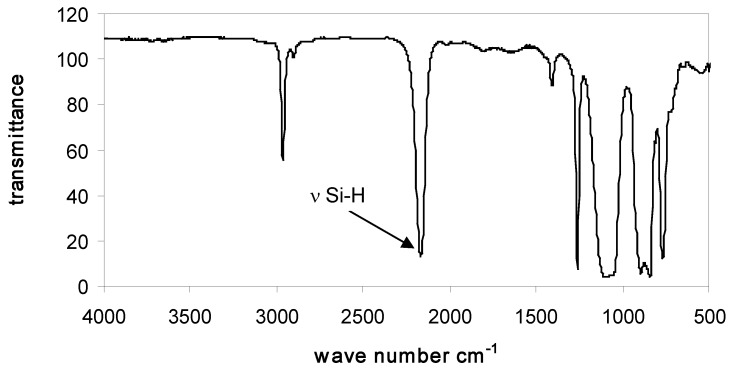
FTIR spectrum of Q_3_D^H^_50_M_8_.

**Figure 3:**
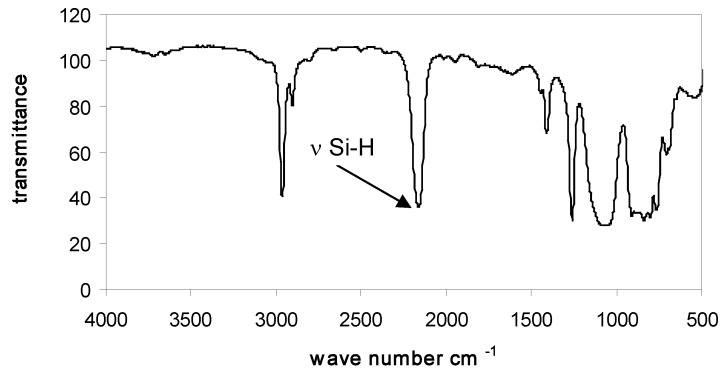
FTIR spectrum of Q_2_D_49_D^H^_49_M_6_.

In all FTIR spectra of studied PMHS-Q containing quadruple branching points Q were present absorption bands in the range 2160 cm^−1^, corresponding to stretching vibrations of Si-H bonds, and also absorption bands of the remaining groups of atoms: Si-CH_3_ (2960, 2890, 1440, 1400, 1255, and 830–700 cm^−1^), Si-O-Si (1010–1110 cm^−1^), and Si(CH_3_)_3_ (750, 690 cm^−1^) (see data in Table 4). Examples of the FTIR spectra of branched poly(methylhydrosiloxanes) are presented in Figure 1, Figure 2 and Figure 3.

### 3.3. Characterization of PMHS-Q by NMR

**Figure 4:**
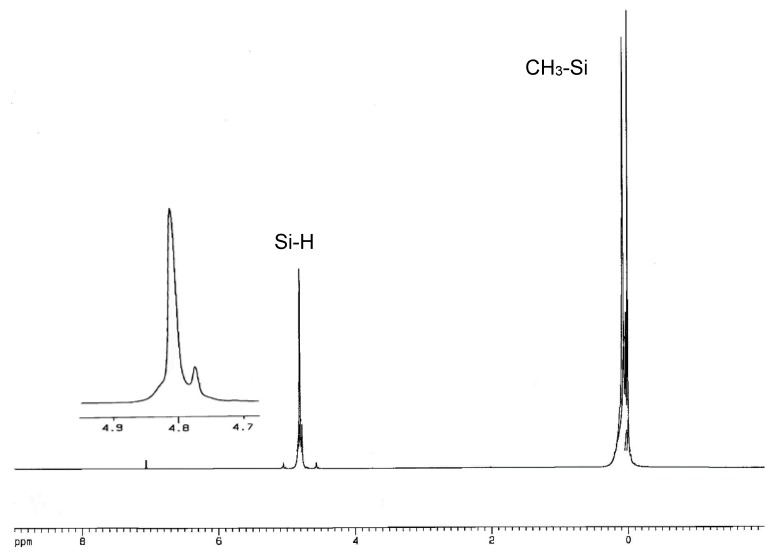
^1^H-NMR spectrum (in C_6_D_6_) of polymethylhydrosiloxane Q_3_D^H^_50_M_8_.

**Figure 5:**
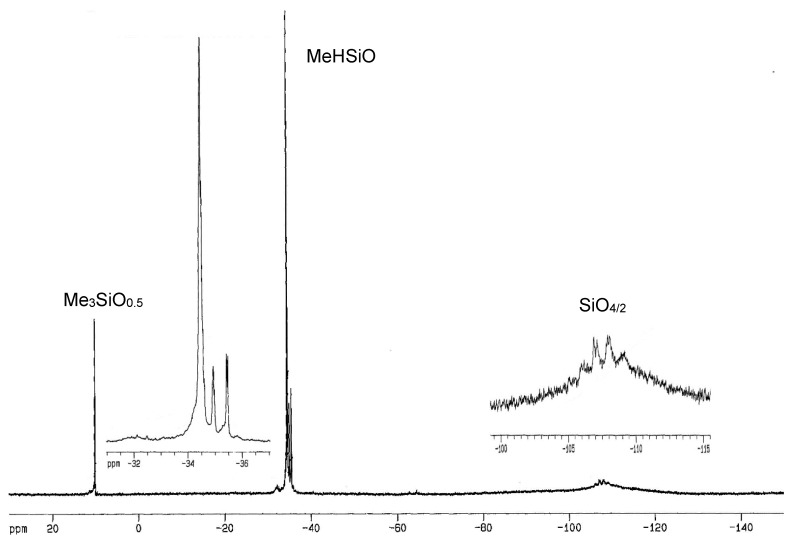
^29^Si-NMR spectrum (INVGATE, in C_6_D_6_) of polymethylhydrosiloxane Q_3_D^H^_50_M_8_.

**Figure 6:**
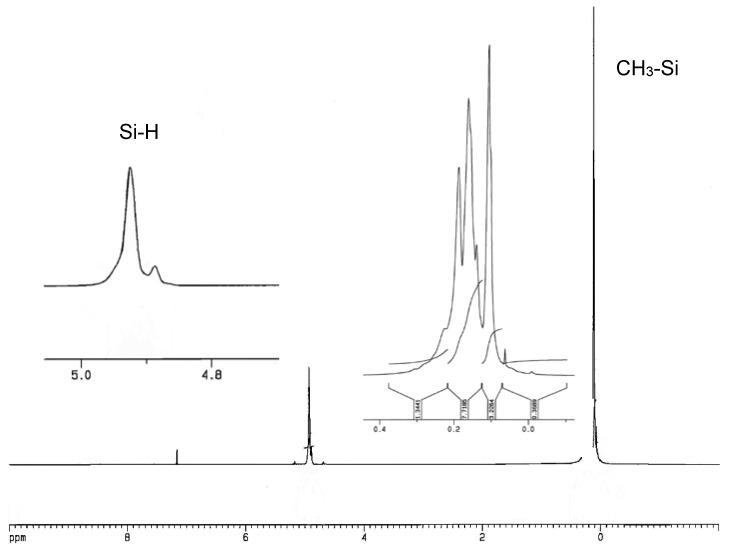
^1^H-NMR spectrum (in C_6_D_6_) of polymethylhydrosiloxane Q_2_D_49_D^H^_49_M_6_.

**Figure 7:**
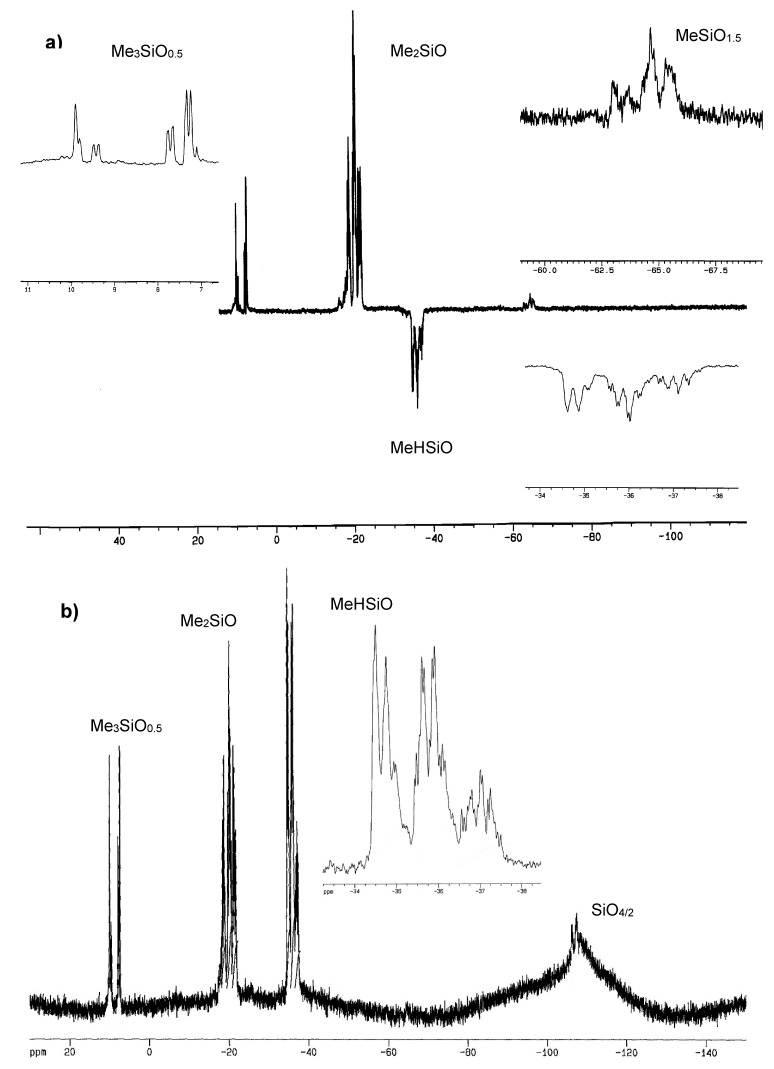
^29^Si-NMR spectra (in C_6_D_6_) of polymethylhydrosiloxane Q_2_D_49_D^H^_49_M_6_: (**a**) INEPT, (**b**) INVGATE.

In ^1^H–NMR spectra of copolymers, **QD^H^_48_M_4_**, **Q_2_D^H^_49_M_6_** and **Q_3_D^H^_50_M_8_** were present signals at δ 0.01–0.22 ppm, corresponding to hydrogen atoms of Si-CH_3_ groups and signals at δ about five parts per million, characteristic for hydrosilane groups Si-H. In the ^1^H-NMR spectra of copolymers: **QD_52_D^H^_52_M_4_**, **Q_2_D_49_D^H^_49_M_6_**, and **Q_3_D_50_D^H^_50_M_8_** were present signals at δ 0.0–0.30 ppm, corresponding to hydrogen atoms of Si–CH_3_ groups and signals at δ about five parts per million, characteristic for Si-H groups. Examples of the ^1^H-NMR and ^29^Si-NMR spectra of branched poly(methylhydrosiloxanes) are presented in Figure 4, Figure 5, Figure 6 and Figure 7.

In ^29^Si-NMR INEPT spectra of copolymers **QD^H^_48_M_4_**, **Q_2_D^H^_49_M_6_** and **Q_3_D^H^_50_M_8_** were present signals at δ −31.62–−39.97 ppm corresponding to silicon atoms of units D^H^ [8,9,54] and at δ 9.40–11.25 ppm corresponding to silicon atoms of end groups Me_3_SiO_0.5_ (M). In the range of δ −63–−68 ppm in INEPT ^29^Si-NMR spectra were present signals of a very low intensity, from Si atoms of branching units MeSiO_1,5_, which could be formed during trace hydrolysis of Si-H bonds. In the INEPT NMR spectra of copolymers **QD_52_D^H^_52_M_4_** (**Q1D**), **Q_2_D_49_D^H^_49_M_6_** (**Q2D**), and **Q_3_D_50_D^H^_50_M_8_** (**Q3D**) existed signals at δ 7.27–9.92 ppm, corresponding to silicon atoms of end groups Me_3_SiO_0.5_ and at δ −34.61 to −38.87 ppm, characteristic for mers MeHSiO (D^H^), and also at δ −18.77–−21.85 ppm from silicon atoms of units Me_2_SiO (D) [8,9,52]. It was impossible to observe signals of quadruple silicon atoms of units SiO_4/2_ in ^29^Si-NMR spectra, which were registered by the INEPT technique, so it was necessary to run ^29^Si–NMR spectra with application of the INVGATE program. A summary of chemical shifts data in the ^1^H- and ^29^Si-NMR (INEPT and INVGATE) spectra of all PMHS-Q is presented in Table 5.

In the ^29^Si-NMR INVGATE spectra of branched random PMHS were present signals of silicon atoms corresponding to linear mers:
CH_3_(H)SiOat δ −34.0–−36.0 ppm (for Q1, Q2, and Q3),
at δ −34.0–−37.5 ppm (for Q1D, Q2D, and Q3D),(CH_3_)_2_SiOat δ −16.5–−22.0 ppm (for Q1D, Q2D, and Q3D),
and terminal groups (CH_3_)_3_SiO_1/2_:

at δ 9.5–11.3 ppm (for Q1, Q2, and Q3),

at δ 7.3–7.9 and 9.8–10.0 ppm (for Q1D, Q2D, and Q3D).

Resonance signals of Si atoms of branching units Q were present in the range of δ:

−100.3–−112.4 ppm,

and they overlapped with very strong ^29^Si signals of a borosilicate glass.

In the ^29^Si-NMR INVGATE spectra of copolymers: **QD^H^_48_M_4_**, **Q_2_D^H^_49_M_6_** and **Q_3_D^H^_50_M_8_** were present signals at: δ 9.40–11.25 ppm, corresponding to the terminal groups Me_3_SiO_0.5_, at δ −31.81–−36.25 ppm, characteristic for units MeHSiO (D^H^), and also resonance signals in the range of δ −100.3–−112.3 ppm for Si atoms from units **Q**. According to data in the literature [73] chemical shifts of Si atoms from units Q exist in the range of δ −100–−106 ppm.

Chemical shifts at δ 9–11 ppm have been assigned to resonances of Si atoms of Me_3_SiO_0.5_ (M) groups in the following sequences of the siloxane chain ends: **M**D^H^D^H^D^H^, **M**D^H^D^H^D, **M**D^H^DD^H^, **M**D^H^DD, **M**D^H^D^H^Q, **M**D^H^QD^H^, **M**D^H^DQ, and** M**D^H^QD, while chemical shifts at δ 7–8 ppm belong to resonances of Si atoms of end groups M in the sequences: **M**DDD, **M**DDD^H^, **M**DD^H^D, **M**DD^H^D^H^, **M**DDQ, **M**DQD, **M**QDD, and **M**QDD^H^. Chemical shifts of middle silicon atoms of units D change in pentades, and magnetic interactions are shifted through four bonds in chain ends.

The sequences of dimethylsiloxane linkages in polymethylhydrosiloxane copolymers might be the following: DD^H^**D**D^H^D, DD^H^**D**D^H^D^H^, D^H^D^H^**D**D^H^D^H^, QD^H^**D**D^H^Q, MD^H^**D**D^H^D, MD^H^**D**D^H^D^H^, MD^H^**D**D^H^Q, MD^H^**D**D^H^D (δ of middle silicon atoms of units **D**: −20–−22 ppm), and: D^H^D**D**D^H^D^H^, D^H^D**D**D^H^D, DD**D**D^H^D^H^, DD**D**D^H^D (δ of middle silicon atoms of units **D**: −18–−19.7 ppm).

In the ^29^Si-NMR spectra (recorded by INEPT and INVGATE techniques) in the range of δ −33–−37 ppm exist signals of middle silicon atoms of units **D^H^**, which undergo changes in pentades (Table 5). Signals of silicon atoms in the range of δ −102 to −109 ppm, presumably correspond to Si atoms in the central units **Q**, in the following sequences of siloxane structures:


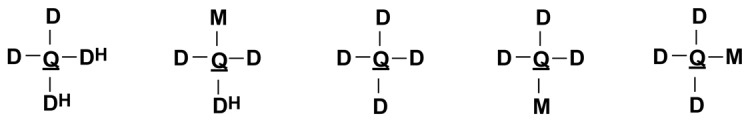


Chemical shifts in the range of 7–11 ppm in the ^29^Si-NMR spectra (INEPT and INVGATE) correspond to silicon atoms of the end groups M and change in tetrads (Table 5) [8,9,74].

Signals at δ −64 ppm of a very low intensity, registered both in INVGATE and INEPT ^29^Si-NMR spectra of these three copolymers, probably come from Si atoms of units MeSiO_1.5_ (T), which were formed during syntheses of PMHS-Q from trace hydrolysis of Si-H bonds [74].

Assignments of all ^29^Si-NMR signals resulting from the microstructure of siloxane chain of branched polymethylhydrosiloxanes are summarized in Table 6.

## Figures and Tables

**Table 1:** Amounts of substrates and a solvent, reaction time in syntheses of branched PMHS containing quadruple branching points, and methods of drying of products solutions.

Substrates, solvent, and reaction conditions		Predicted molecular formulas of PMHS-Q *					
		QD^H^_48_M_4_	Q_2_D^H^_49_M_6_	Q_3_D^H^_50_M_8_	QD_52_D^H^_52_M_4_	Q_2_D_49_D^H^_49_M_6_	Q_3_D_50_D^H^_50_M_8_
		Amounts of reagents and a solvent					
**Si(OEt)_4_**	(mol)	0.01	0.04	0.06	0.01	0.02	0.03
	(cm^3^)	2.2	8.9	13.4	2.2	4.6	6.7
**MeHSiCl_2_ (MDS)**	(mol)	0.48	0.98	1.0	0.52	0.49	0.50
	(cm^3^)	50.0	102.0	104.1	54.1	51.5	52.1
**Me_2_SiCl_2_ (DDS)**	(mol)	-	-	-	0.52	0.49	0.50
	(cm^3^)	-	-	-	63.1	59.8	60.6
**Me_3_SiCl (TMCS)**	(mol)	0.04	0.12	0.16	0.04	0.06	0.08
	(cm^3^)	5.1	15.2	20.3	5.1	7.6	10.2
**H_2_O**	(mol)	9.11	18.94	19.66	19.33	18.38	18.94
	(cm^3^)	164	341	354	348	331	341
**(4-dimethylamino)-pyridine (DMAP)**	(mol)	-	-	0.003	0.0109	0.0108	0.01
	(g)	-	-	0.3665	1.3316	1.3194	1.2217
**Et_3_N**	(mol)	-	-	0.03	0.109	0.108	0.10
	(cm^3^)	-	-	4.2	101.2	15.1	13.9
Diethyl ether	(cm^3^)	50	80	60	100	80	90
Addition time of chlorosilanes and Si(OEt)_4_	(min)	50	60	95	80	55	95
Temperature during addition of chlorosilanes and Si(OEt)_4_	(°C)	−4–−2	−1–3	−1–3	−1–3	−1–6	−2–2
Stirring time after addition of chlorosilanes and Si(OEt)_4_	(min)	170	130	130	130	120	120
Drying of products:		with anhydrous MgSO_4_			by cooling in a fridge		

* Average molecular composition of polymers, based on a stoichiometry of monomers. PMHS-Q: liquid branched poly(methylhydrosiloxanes) of random structures.

**Table 2:** Yields of PMHS-Q, conditions of removal of volatile products, and results of measurements of dynamic viscosity of branched PMHS, containing quadruple branching units Q.

PMHS-Q			Dynamic viscosity	Evacuation conditions		Volatile products	
Predicted molecular formula (polymer abreviation)	Yield			Bath temp.	Time	B.p./pressure	Mass
	(g)	(wt%)	(cP)	(°C)	(min)	(°C/mm Hg)	(g)
**QD^H^_48_M_4_ (Q1)**	20.14	62	12.8	152	190	24/16–79/3.5	9.27
**Q_2_D^H^_49_M_6_ (Q2)**	48.70	68	11.0	154	200	23/16–77/3.5	18.14
**Q_3_D^H^_50_M_8_ (Q3)**	52.82	69	13.1	155	190	23/18–70/3.5	16.71
**QD_52_D^H^_52_M_4_ (Q1D)**	40.29	55	12.5	150	210	21/21–80/5	34.84
**Q_2_D_49_D^H^_49_M_6_ (Q2D)**	40.49	56	11.8	155	200	27/19–78/4	31.06
**Q_3_D_50_D^H^_50_M_8_ (Q3D)**	43.77	58	10.7	155	190	21/16–74/4.5	28.22

**Table 3:** Characteristics of polymethylhydrosiloxanes with branched, random structure of siloxane chains, containing branching units Q.

PMHS-Q	M_n_ (calc.)	M_n_	M_w_	M_w_/M_n_	% C		% H		% Si	
					calc.	found	calc.	found	calc.	found
**QD^H^_48_M_4_**	3271	6310	17,750	2.81	22.03	21.51 21.76	7.03	6.61 6.90	45.50	44.87
**Q_2_D^H^_49_M_6_**	3554	3220	8330	2.59	22.64	22.01 22.11	7.09	6.92 7.01	45.05	45.15
**Q_3_D^H^_50_M_8_**	3836	3840	10,350	2.69	23.16	22.03 22.14	7.14	7.02 6.85	44.65	43.81
**QD_52_D^H^_52_M_4_**	7367	2650	6280	2.36	27.39	28.89 28.70	7.60	7.89 8.03	41.55	41.54
**Q_2_D_49_D^H^_49_M_6_**	7187	2440	5750	2.35	27.57	28.15 28.40	7.63	7.69 7.88	41.42	41.40
**Q_3_D_50_D^H^_50_M_8_**	7544	5100	10,210	2.00	27.07	27.78 27.92	7.65	8.35 7.98	41.32	41.30

**Table 4:** Characteristic absorption bands in FTIR spectra of Q_2_D_49_D^H^_49_M_6_.

Wave number [cm^−1^]		Group or bond	Vibration
found	literature data [72]		
2965	2975–2950	CH_3_	ν _asym_ C-H
2878	2885–2860	CH_3_	ν _sym_ C-H
2164	2300–2100	Si-H	ν Si-H
1450	1470–1420	CH_3_	δ _asym_ C-H
1410	1390–1365	CH_3_	δ _sym_ C-H
1260	1265–1250	Si-CH_3_	δ _asym_ Si-C
1115–1027	1100–1000	Si-O-Si	ν _asym_ Si-O
910	950–800	Si-H	δ Si-H
864	860–750	Si-CH_3_	ν _asym_ Si-C
830	910–830	Si-O	ν _asym_ Si-O
800	800	Si-CH_3_	δ _sym_ Si-C
759	755	Si-(CH_3_)_3_	δ _asym_ Si-C

**Table 5:** Chemical shifts of PMHS-Q in their ^1^H- and ^29^Si-NMR spectra (in C6D6).

PMHS-Q	δ (ppm)										
	^1^H-NMR		^29^Si-NMR								
			INEPT	INVGATE	INEPT	INVGATE	INEPT	INVGATE	INEPT	INVGATE	
	Si–H	Si–CH_3_	Me_3_SiO_0.5_		Me_2_SiO		MeHSiO		MeSiO_3/2_		SiO_4/2_
**QD^H^_48_M_4_**	4.90	0.01–0.22	9.75–11.03	9.98–10.96	-	-	−31.51–−37.42	−31.85–−35.90	−64.51	−64.58	−101.40–−108.13
**Q_2_D^H^_49_M_6_**	4.80	0.01–0.12	9.40–11.24	9.70–10.32	-	-	−31.62–−39.97	−31.83–−36.25	−62.60–−64.61	-	−101.37–−112.33
**Q_3_D^H^_50_M_8_**	4.80	0.01–0.12	9.40–11.25	9.42–11.28	-	-	−37.46–−31.16	−32.16–−35.87	−62.97–−64.56	−64.54	−100.31–−110.20
**QD_52_D^H^_52_M_4_**	4.92	0.05–0.22	9.92–7.27	7.30–9.96	−18.48–−21.74	−18.67–−21.44	−33.27–−38.87	−34.56–−37.30	−63.12–−65.39	−63.72–−65.89	−102.92–−109.92
**Q_2_D_49_D^H^_49_M_6_**	4.92	0.01–0.30	9.92–7.26	7.32–9.98	−18.43–−21.85	−18.70–−21.69	−34.54–−37.33	−34.61–−37.03	−62.95–−65.26	−62.35–−66.16	−101.81–−109.71
**Q_3_D_50_D^H^_50_M_8_**	4.83	0.01–0.23	9.91–7.27	7.32–9.98	−18.77–−21.75	−18.72–−21.29	−34.61–−37.57	−34.54–−36.10	−64.73	−63.35–−65.16	−102.84–−109.61

**Table 6:** The microstructure of siloxane chains in PMHS-Q, containing quadruple branching units Q (all possible sequences among tetrads of terminal groups, and linear and star pentads); values of δ concern Si atoms in structural units denoted as bold and underlined.

δ ^29^Si-NMR (ppm)					
9–11	7–8	−18–−19	−20–−22	−33–−37	−101–−109
**M**D^H^D^H^D^H^ **M**D^H^D^H^Q **M**D^H^QD^H^ **M**D^H^D^H^D **M**D^H^D^H^D^H^ **M**D^H^DD^H^ **M**D^H^DD	**M**DDD **M**DDD^H^ **M**DD^H^D **M**DD^H^D^H^ **M**DDQ **M**DQD **M**QDD **M**QDD^H^	DD^H^**D**D^H^D DD^H^**D**D^H^D^H^ D^H^D^H^**D**D^H^D^H^ DD^H^**D**D^H^Q QD^H^**D**D^H^Q MD^H^**D**D^H^D MD^H^**D**D^H^D^H^ MD^H^**D**D^H^Q MD^H^**D**D^H^D	D^H^D**D**D^H^D^H^ D^H^D**D**D^H^D DD**D**D^H^D^H^ DD**D**D^H^D D^H^D**D**DQ D^H^D**D**QD DD^H^**D**DQ DD^H^**D**QD DD**D**DQ DD**D**QD QD**D**DQ QD**D**QD DQ**D**QD DQ**D**DQ MD**D**DQ MD**D**QD MQ**D**DD MD**D**DQ MQ**D**QD	D^H^D^H^**D^H^**D^H^D^H^ DD^H^**D^H^**D^H^D^H^ D^H^D**D^H^**D^H^D^H^ DD^H^**D^H^**D^H^D DD^H^**D^H^**DD^H^ D^H^D**D^H^**D D^H^ D^H^D**D^H^**D^H^Q D^H^D^H^**D^H^**QD^H^ DD^H^**D^H^**D^H^Q D^H^D**D^H^**D^H^Q D^H^D**D^H^**QD^H^ DD^H^**D^H^**^H^QD^H^ DD**D^H^**QD^H^ D^H^D^H^**D^H^**DQ D^H^D^H^**D^H^**QD DD^H^**D^H^**DQ DD^H^**D^H^**QD D^H^D**D^H^**DQ D^H^D**D^H^**QD DD**D^H^**D^H^Q DD**D^H^**DQ MD^H^**D^H^**D^H^D^H^ MD^H^**D^H^**D^H^ D MD^H^**D^H^**DD^H^ MD**D^H^**D^H^D^H^ MD**D^H^**D^H^D MD**D^H^**DD^H^ MD^H^**D**^H^D^H^Q MD**D^H^**D^H^Q MD^H^**D^H^**DQ MD^H^**D^H^**QD MD^H^**D^H^**QD^H^ MD**D^H^**QD^H^ MD**D^H^**DQ	D D**Q**D D D D**Q**D M D D**Q**M M D D**Q**D^H^ D D D**Q**D^H^ D^H^ M D**Q**D D^H^
